# *In vitro* Assessment of the Safety and Potential Probiotic Characteristics of Three *Bacillus* Strains Isolated From the Intestine of Hybrid Grouper (*Epinephelus fuscoguttatus*♀ × *Epinephelus lanceolatus*♂)

**DOI:** 10.3389/fvets.2021.675962

**Published:** 2021-05-28

**Authors:** Kwaku Amoah, Xiao-hui Dong, Bei-ping Tan, Shuang Zhang, Felix K. A. Kuebutornye, Shu-yan Chi, Qi-hui Yang, Hong-yu Liu, Hai-tao Zhang, Yuan-zhi Yang

**Affiliations:** ^1^Laboratory of Aquatic Animal Nutrition and Feed, College of Fisheries, Guangdong Ocean University, Zhanjiang, China; ^2^Aquatic Animals Precision Nutrition and High-Efficiency Feed Engineering Research Centre of Guangdong Province, Zhanjiang, China; ^3^Key Laboratory of Aquatic, Livestock and Poultry Feed Science and Technology in South China, Ministry of Agriculture, Zhanjiang, China; ^4^Provincial Key Laboratory of Pathogenic Biology and Epidemiology for Aquatic Animals, Zhanjiang, China

**Keywords:** *Bacillus tequilensis*, *Bacillus velezensis*, *Bacillus subtilis*, probiotics, hybrid grouper (*Epinephelus fuscoguttatus* ♀ × *Epinephelus lanceolatus* ♂), antibiotic resistance

## Abstract

Probiotics serving as an alternative to the criticized antibiotics mainly focus on improving animal's growth and health. After realizing the dangers posed by diseases that have led to lots of economic losses, aquaculture scientists have sought the usage of probiotics. However, most probiotics are ineffective in eliciting aquatic animals' preferred effects, since they are from non-fish sources. Again, there are even a few marine aquatic probiotics. Given this, a study was conducted to investigate the probiotic potential of the bacteria species isolated from the digestive tract of hybrid grouper (*Epinephelus fuscoguttatus*♀ × *Epinephelus lanceolatus*♂). Based on the morphological, biochemical, 16S rRNA sequencing analysis and evolutionary relationships, the isolated species were identified as *Bacillus tequilensis* GPSAK2 (MW548630), *Bacillus velezensis* GPSAK4 (MW548635), and *Bacillus subtilis* GPSAK9 (MW548634), which were designated as GPSAK2, GPSAK4, and GPSAK9 strains, respectively. Their probiotic potentials including their ability to tolerate high bile salt concentration, low pH, high temperatures, adhesion ability (auto-aggregation and cell-surface hydrophobicity), antimicrobial activity and biosafety test, compatibility test, hemolytic activity, and antibiotic susceptibility test were evaluated. While GPSAK2 and GPSAK9 strains were γ-hemolytic, that of GPSAK4 was α-hemolytic. All the isolates were resistant to low pH (1) and higher bile salt concentration (0.5%), showed higher viability ability after higher temperature exposure (80, 90, and 100°C), as well as higher cell-surface percentage hydrophobicity and auto-aggregation. All isolates exhibited positive compatibility with each other, signifying their ability to be used as multispecies. The three strains were susceptible to ampicillin (except GPSAK9, which was resistant), penicillin, kanamycin, ceftriaxone, chloramphenicol, erythromycin, clindamycin, furazolidone (except GPSAK2 and GPSAK9, which were moderately susceptible and resistant, respectively), polymyxin B, vancomycin (except GPSAK9, which was resistant), sulfamethoxazole (except GPSAK9, which was moderately susceptible), amikacin, minocycline, ofloxacin, norfloxacin, doxycycline, neomycin, gentamicin, tetracycline, carbenicillin, midecamycin (except GPSAK9, which was moderately susceptible), ciprofloxacin, piperacillin, and cefoperazone. All isolates demonstrated good antimicrobial activity against four pathogens, *viz. Streptococcus agalactiae, Streptococcus iniae, Vibrio harveyi*, and *Vibrio alginolyticus*. The results collectively suggest that *Bacillus* strains GPSAK2, GPSAK4, and GPSAK9 could serve as potential probiotic candidates that can be used to improve the growth and health status of aquatic animals, especially grouper.

## Introduction

With the incessant pressure on governments and the international communities in ensuring sufficient food supply for the ever-growing population, aquaculture has emerged as one of the promising industries to fight this global food insecurity crisis, since it provides food at a relatively cheaper price without sacrificing its nutritional value. Food and Agriculture Organization (1) in its report stated that the global production for aquaculture, which was 73.8 million tons (MT) (first sale estimated at $160.2 billion) in 2014, increased to 110.2 MT (first sale estimated at $243.5 billion) in 2016, signifying how the populace has accepted aquaculture food. Hulong grouper, a novel hybrid of brown-marbled and giant grouper (*Epinephelus fuscoguttatus*♀ × *Epinephelus lanceolatus*♂), is a new fish species first produced in 2007 by the Universiti Malaysia Sabah (2) and currently noted as one of the most economically valuable grouper species farmed in Southeast Asia. It is a typical carnivores species that is a perfect candidate species for highly intensive aquaculture due to its ability to withstand high population density, faster growth, high disease resistance, efficient feed conversion (3, 4), and high nutritional and economic value (5, 6). Nonetheless, the rapid development of this fish's intensive and super-intensive culture has led to diverse incidences of poor growth performance and disease infections (7). For example, while Qin et al. (8) revealed the infection of iridovirus in the greasy grouper (*E*. *tauvina*), Shen et al. (9) in their work also reported that the causal agent of skin ulcer disease in juvenile hybrid grouper (*E*. *fuscoguttatus*♀ × *E*. *lanceolatus*♂) was *Vibrio harveyi*. This high infection rate has resulted in higher economic losses in the grouper farms and hatcheries. Farmers have adopted vaccines, antibiotics, and other prophylactic control mechanisms to help control such diseases, but their usage has brought numerous side effects (10, 11). Antibiotic usage, for example, has been criticized, since they cause serious ecological and biological effects, as they have led to the emergence of antibiotic-resistant bacteria and genes, as well as residual antibiotics in cultured organisms posing health risks to animals and humans (12–14). Reports have it that there are at least 2.8 million people suffering from serious infections with antibiotic-resistant bacteria, which result in a yearly death of at least 35,000 people in the United States (15), suggesting the need for a quick response in combating such threat.

Probiotics, which are defined as “live microorganisms which, when administered in adequate amounts, confer a health benefit to the host” (1), are currently proposed as the effective and eco-friendly alternative to antibiotics (16) due to (i) their antagonistic activities against pathogenic bacteria (17, 18), which is generally because of their ability to secrete bacteriocins (19) and other compounds; (ii) their ability to alleviate allergic symptoms and inflammation; and (iii) their ability to improve growth and keep a positive balance of intestinal microbial composition (20, 21). Grand View Research in its work has reported that the global market size of probiotics was estimated at $48.38 billion in 2018 and is projected to reach $77.09 billion by 2025 with a compound annual growth rate (CAGR) of 6.9% (https://www.grandviewresearch.com/industry-analysis/probiotics-market), and this calls for more research to be conducted to unearth new strains of importance to meet the target. Given the significance of probiotics in maintaining the health of fish, with respect to their involvement on immunocompetence and disease resistance, in addition to its role in stress mediation, there is a growing trend toward exploring new species and strains with more endowed features as a novel probiotic candidate (22). The digestive tract of fish offers an enabling environment for the growth and survival of bacteria, which aids the bacterial community in exhibiting numerous enzymatic potentials, which successively helps in digestion (23, 24). The digestive tract microorganisms are noted to synthesize numerous enzymes such as lipolytic, proteolytic, amylolytic, and cellulolytic enzymes involved in the digestion of lipids, proteins, carbohydrates, and cellulose, respectively (25, 26). Nonetheless, the production of the above-listed enzymes is premised on the bacteria's ability to survive in the gastrointestinal condition, including resisting low pH and gastric juice (27, 28). Again, for the usage of an identified probiotic in feed making, the bacteria should have the ability to withstand high temperatures to stay viable after feed processing, since most food processes require heat (29). Bacteria positive for hemolysis are regarded as unsafe for use as probiotics due to their virulence causing edema and anemia. Also, a chosen probiotic bacterium should be able to synthesize bacteriocins that aid in the inhibition of pathogens, thus, for sustainable aquaculture operations, the screening and selection of the probiotics to be used should be key (30).

Among the several probiotics discovered, *Bacillus* spp. have over the years proven to be one of the most commonly applied probiotics in aquaculture production due to their enormous attributes such as their ability to stay viable (sporulation capacity) for a more extended period in harsh conditions (heat and pH tolerant), ability to produce non-pathogenic and non-toxic compounds but instead produce a massive range of extracellular substances including lipase, amylase, trypsin, and antimicrobial peptide (20, 31–35), which thereby translates into proving positive results for growth and health enhancement, as well as disease resistance in animals and humans. Again, among the various sources of *Bacillus* spp. that include water, soil, decaying matter, and other commercial sources (36–38), those isolated from the fish intestinal tract are more effective on their host compared to others (39–42). Hence, the isolation of probiotics from fish will be commendable in enhancing aquaculture development.

Accordingly, this work's objective was to isolate potential *Bacillus* strains from the intestine of hybrid grouper (*E*. *fuscoguttatus*♀ × *E*. *lanceolatus*♂). For that reason, we have isolated three *Bacillus* species, and their probiotic properties are characterized based on their safety to host, their benefits to host (biofilm formation, cellulase production, non-hemolytic nature), and their abilities to be used as feed (sporulation, resistance to heat, low pH, bile tolerance, adhesions to epithelial cells), and we hope for its usage as potential probiotics and immunoadjuvant for fish culture.

## Materials and Methods

### Animal Sample Collection

Healthy samples of hybrid grouper (*E*. *fuscoguttatus*♀ × *E*. *lanceolatus*♂) without symptoms of infection [i.e., gross examination of hemorrhage, edema, lethargic, lesions, and detachment of scales (43)] with an average weight of 85 ± 2.77 g were obtained from a local farm situated close to the South China sea (Zhanjiang, Guangdong Province, China) and kept in aerated tanks that were later transported to the laboratory for the immediate commencement of the experiment.

### Strain Isolation and Identification

#### Strain Isolation and Growth Conditions

Fish were anesthetized by immersion in a tank containing tricaine methane-sulfonate (MS-222; Sigma-Aldrich) at 150 mg/L and then killed by a blow to the head. With the help of cotton dipped into 75% ethanol, fish were cleaned externally to remove or kill bacteria on their bodies to avoid bacterial contamination. Under sterile conditions, the fish guts were dissected using sterile scissors and tweezers, stripped carefully to remove all digesta content, and washed using a sterile physiological saline solution (PSS). The intestinal weight was taken, and equal proportions of PSS by volume were added. Under sterile conditions, the gut content was then homogenized using 15 ml borosilicate glass tissue homogenizer (Shanghai Lenggu Instrument Company, Shanghai, China) in ice, of which 0.5 ml of the gut homogenate was diluted with 5 ml PSS. The mixture was serially diluted using PSS, and 0.1 ml aliquot was spread on Luria–Bertani (LB) (Beijing Land Bridge Tech. Co., Ltd.) agar plates followed by incubation at 30°C for 24 h. Discrete bacterial colonies were randomly picked and inoculated into LB media for mass culture under the same culture conditions but with agitation at 180 rpm/min. Streaking of the isolates was repeatedly done to obtain very pure colonies. All the experiments, including this part, were conducted under sterile conditions.

#### Strain Identification

Potential probiotic strains were characterized based on their morphology, biochemical tests, antimicrobial tests against some pathogenic bacteria, and antibiotic resistance and identified by molecular 16S rRNA gene sequence analysis using universal bacterial primers 27F (AGAGTTTGATCCTG GCTCAG) and 1492R (GGTTACCTTGTTACGACTT) through polymerase chain reaction (PCR) (44). The PCR reaction system contained 1 μl template of each isolate genomic DNA, 1 μl of each primer, 12.5 μl of 10 × Ex*taq* buffer, and 9.5 μl of double-distilled water (ddH_2_O). For negative control, ddH_2_O was used as a template, whereas that of the positive control was a previously isolated bacteria from *V. harveyi* (45). The PCR amplification was initiated with denaturation at 96°C for 5 min followed by 33 cycles of denaturation at 96°C for 30 s, annealing at 55°C for 45 s, and extension at 72°C for 1 min 30 s; the amplification was completed by holding the reaction mixture at 72°C for 10 min. The PCR products were analyzed by agarose gel (1% w/v) electrophoresis. Amplicons were eluted, and the purified DNA products were later sent to Sangon Biotech Co., Ltd. (Guangzhou, China) for sequencing. The nucleotide sequences were subsequently compared with the available sequences in the database of the National Center for Biotechnology Information (NCBI) using Basic Local Alignment Search Tool (BLASTn) program. Similarity analysis was conducted to best identify the probiotic strain types. The phylogenetic tree was generated by neighbor-joining (NJ) method using NCBI Distance tree online tool. All selected strains were stored in equivalent mixed solution of 40% (*v/v*) glycerol and LB until use.

### Characterization of Isolated Strains Based on Biochemical Tests

Biochemical characterization tests are conducted to examine the agreement of genetic analysis and phylogenetic studies of the isolates. As illustrated in **Table 2**, the selected probiotic strains' biochemical characterization was performed using commercial kits procured from Guangdong Huankai Microbial Sci. and Tech. Co., Ltd. (Guangdong, China) following the company's instruction. With the help of a *Bacillus cereus* identification bar (HBIG07-1) purchased from the Qingdao Hope Bio-Tech. Co., Ltd. (Qingdao, China), the biochemical tests conducted were confirmed.

### Growth Pattern of the Selected Strains in Luria–Bertani Broth

A selected single colony of probiotic bacteria strain from LB agar (pH 7.2) was first incubated overnight in 5 ml LB broth (37°C). One (1) milliliter of the overnight culture was inoculated into 100 ml LB broth (pH 7.2) in 500-ml Erlenmeyer flasks of which were incubated in a shaken incubator (150 rpm/min at 37°C for 24 h). During the culturing period, the bacteria growth was measured at 2-h intervals until reaching the 24-h stipulated time using a spectrophotometer (Evolution™ 220 UV-Visible Spectrophotometer, Thermo Scientific™, USA) at an absorbance optical density (OD) of 600 nm (46). Finally, the growth curve of the different strains was plotted. All the experiments were repeated in triplicate with the reading of the profiles being averaged.

### Biosafety Test

This experiment was conducted to ascertain the possible harmful effects of the selected probiotic strains in healthy hybrid grouper. Healthy hybrid grouper fish (*E*. *fuscoguttatus*♀ × *E*. *lanceolatus*♂) weighing 90–100 g were purchased from a local fish farm (Zhanjiang, China). They were thus maintained in aerated cement pools [4.5 m (l) × 3.45 m (w) × 1.8 m (h)] for an acclimatization period of 2 weeks and were daily hand-fed twice (08:00 and 16:30) at 5% of their body weight with commercial diets (procured from Zhanjiang Aohua Feed Co. Ltd., Guangdong, China). After adaptation, a total of 90 fish (average weight 97 ± 1.34 g) after 24-h starvation were weighed and randomly distributed into nine fiberglass tanks (0.3 m^3^) at 10 fish-density per tank (i.e., divided into three groups for the three isolated probiotic strains (GPSAK2, GPSAK9, and GPSAK4 groups) with three replications each. The biosafety experiment, which lasted 21 days, was conducted in an indoor facility of the Marine Biological Research Base of Guangdong Ocean University (situated close to the South China Sea) under a photoperiod of natural 12-h light/12-h dark regime with a 2-day interval of 50% water exchange. Single airstones provided aeration, and the water quality of dissolved oxygen, temperature, pH, and salinity maintained as ≥6 mg L^−1^, 28–30°C, 7.8–8.2, and 28.5–32%, respectively (YSI 556 multiprobe system, YSI Inc., USA). The isolates' suspension was prepared as stated above (see section Growth Pattern of the Selected Strains in Luria–Bertani Broth). Afterward, 0.1 ml [10^8^ colony-forming unit (CFU)/ml] of each isolated bacteria was injected intraperitoneally into their respective grouped fish. An additional 30 fish were kept in three different fiberglass tanks (10 fish/tank) of which were injected with the same volume of sterile phosphate-buffered saline (PBS) (pH 7.2) to serve as the control group. The steps used in acquiring the isolated bacteria's concentration (i.e., 10^8^ CFU/ml) followed a previously described process (20). Fish were then monitored daily to ascertain whether there were any clinical signs, and their mortality was recorded until the 21st day.

### Antibiotic Susceptibility Test

The susceptibility of the isolated *Bacillus* strains was assessed by the disc diffusion method against 24 antibiotics including ampicillin (10 μg), penicillin (10 μg), kanamycin (30 μg), ceftriaxone (30 μg), chloramphenicol (30 μg), erythromycin (15 μg), clindamycin (2 μg), furazolidone (300 μg), polymyxin B (300 μg), vancomycin (30 μg), sulfamethoxazole (27.5 μg), amikacin (30 μg), minocycline (30 μg), ofloxacin (5 μg), norfloxacin (10 μg), doxycycline (30 μg), neomycin (30 μg), gentamicin (10 μg), tetracycline (30 μg), carbenicillin (100 μg), midecamycin (30 μg), ciprofloxacin (5 μg), piperacillin (100 μg), and cefoperazone (75 μg) (Hangzhou Microbial Reagent Co., Ltd., Hangzhou, China). Briefly, 0.1 ml (at an OD adjusted to No. 0.5 McFarland standard 1.5 × 10^8^ CFU/ml) of cultured isolated probiotics was spread on 20 ml Mueller–Hinton agar (Beijing Land Bridge Tech. Co., Ltd.) plates. Subsequently, the antibiotic plates were carefully placed on the surface of the agar plates and incubated at 37°C for 24 h. After incubation, the probiotics' susceptibility was analyzed by measuring (mm) the zone of inhibition as described previously (47).

### Bile Salt Tolerance

The bile salt tolerance was determined following the modified methods of Argyri et al. (48). Tolerance was examined by checking bacterial growth. In brief, bacteria cells from overnight culture after harvesting (9,000 × g for 5 min at 4°C) were washed twice with PBS (pH 7.4) and resuspended in 10 ml PBS (pH 7.4) containing 0.5% (*w/v*) of bile salts (Sangong Biotech Co., Ltd., Shanghai, China). Additional overnight cultures of isolates after harvesting and washing that served as the control were resuspended in PBS void of bile salt. Subsequently, 0.1 ml of the control and the exposed (after 0.5% bile salt exposure) bacteria at different time points (1, 2, 3, and 4 h) were taken and spread onto LB agar plates which was later incubated for 12 h. Viable colonies of the isolates that survived with or without 0.5% bile salt exposure were counted, and the percentage survival of isolates was calculated using the following formula: survival (%) = (B_t_/B_0_) × 100, where B_t_ represented the viable counts or the number of survived cells after incubation when bacterial isolates were exposed to PBS with 0.5% bile salt for 1, 2, 3, or 4 h, and B_0_ is the viable counts or number of survived cells obtained after incubation when bacterial isolates were exposed to PBS with no bile salt (control). Evaluation of the isolated strains' tolerance to bile salt was conducted in triplicate.

### High-Temperature Resistance Test

Following Guo et al. (29) methodology with slight modification, the isolated bacterial strains' resistive capacity to different temperatures was evaluated. Since the processing of fish feed and other animal feeds at times requires high temperature, it is essential to know the isolated bacteria's resistive capacity to understand their survival in such harsh conditions. In doing this, the isolates' overnight cultures, after washing twice with 40 ml PBS (pH 7.4), were then exposed to 80, 90, and 100°C temperature using the Med-L-Hh 6 Electrothermal Thermostatic water-bath heater (Guangzhou Med Equipment Co. Ltd.) for 2, 5, and 10 min. Subsequently, an equal volume of LB broth was added to the heat-treated isolates to determine their ability to grow after treatment with heat. Growth was observed by measuring the absorbance at 600 nm after 12 h of incubation (37°C) with continuous shaking at 150 rpm/min. The high temperature-resistant assay was performed in triplicate.

### Compatibility of the Three Isolates

Following the methodologies of Rajyalakshmi et al. (49), the test of compatibility of the three isolates was conducted. Briefly, the three probiotic isolates were vertically streaked on an LB agar plate 5 mm apart, followed by a perpendicular streaking of 10 mm apart from each other. The plates were afterward incubated (24 h at 37°C), and the compatibility was assessed by observing the zone of inhibition among the isolates.

### Antimicrobial Properties

The selected *Bacillus* strains were evaluated for antimicrobial activity against four pathogenic bacteria, namely; *Streptococcus agalactiae, S*. iniae, *Vibrio harveyi*, and *V. alginolyticus*, which were provided by the Guangdong Key Laboratory of Control for Diseases of Aquatic Economic Animals, Zhanjiang, China. The culturing process and the concentration of the disease bacteria used followed our previously described procedure (20). Briefly, 0.1 ml of each disease bacterium was added to 100 ml of LB in a 250-ml Erlenmeyer flask and cultured in a shaken incubator (180 rpm for 16 h at 37°C). After obtaining the cell pellets through centrifugation (4,930 × g at 4°C for 10 min), they were washed twice using PBS, and their concentration was adjusted at 600 nm wavelength. Through the serial dilution and spread plate technique, the supernatants were resuspended in PBS to get graded doses (10^6^, 10^7^, 10^8^, and 10^9^ CFU/ml). A prior cross streaking and agar well-diffusion experiment was conducted using the graded doses (three repeats each) with the isolated strains to determine which of the concentrations was best for the experiment, and 1 × 10^8^ CFU/ml was chosen. Finally, following the cross streaking and agar well-diffusion method of (50) using the chosen concentration, the pathogens were tested against the selected *Bacillus* strains isolated. The antimicrobial experiment was conducted in triplicate.

### Screening of *Bacillus* Strains for Auto-Aggregation and Cell Surface Hydrophobicity Properties

Auto-aggregation assays were performed as defined by Shin et al. (51) with slight modification. Briefly, *Bacillus* strains were grown overnight at 37°C in LB broth. Their bacterial cells were centrifuged (9,000 × *g*, 3 min), washed twice with sterile PBS (pH, 7.2), resuspended in the supernatant, and then vortexed for 30 s. The absorbance was measured at different times (0, 1, 2, 3, and 24 h) using a UV/visible spectrophotometer at a wavelength of 600 nm. The auto-aggregation percentage was calculated using the following formula: auto-aggregation (%) = (1–[A_t_/A_0_]) × 100, where A_t_ represented the absorbance at time *t* = 1, 2, 3, or 4, and A_0_ represented the absorbance at *t* = 0 h.

The degree of cell surface hydrophobicity of the carefully chosen *Bacillus* strains was determined by employing the method described by Lee et al. (52) based on adhesion of cells to organic solvents with slight modification. Briefly, bacteria from a 24-h culture were harvested by centrifugation (9,400 × *g*, 3 min), washed twice with PBS (pH 7.2), and resuspended in 5 ml of the same buffer. The absorbance of the cell suspension was measured at 600 nm and used as the value A_0_ to determine hydrophobicity (%). The cell suspension was then mixed with the same volume of solvent and mixed thoroughly by vortexing for 5 min, wherein ethyl acetate (a basic solvent), chloroform (an acidic solvent), and xylene (a non-polar solvent) were used. The suspension was incubated at room temperature for 30 min to allow two-phase separation. The aqueous phase removed was read at an absorbance wavelength of 600 nm and was subsequently labeled A_t_. The percentage of bacterial cell adhesion to solvent was calculated using the following formula: hydrophobicity (%) = 1– (A_t_/A_0_) × 100, where A_t_ represented the absorbance of the aqueous phase after the two-phase separation, whereas that of the A_0_ represented the absorbance before mixing with solvent.

### Hemolytic Activity

The three isolated *Bacillus* species were subjected to a hemolytic test by streaking them onto agar plates supplemented with 7% sheep blood. The hemolytic zones were observed after incubation of plates at 37°C for 48 h. The isolates were then classified as α-, β-, and γ-hemolysis. The isolates having a green zone around the colony were recorded as α-hemolysis, while those with a clear zone were designated as β-hemolysis. Also, those that did not produce any zone around the colony were referred to as no- or γ-hemolysis (52, 53).

### Determination of Optimal Growth and pH of the Three Isolated Probiotics

The optimal growth and pH were evaluated in conformity to Kavitha et al. (33). Briefly, the three bacterial isolates' fresh overnight cultures were inoculated in LB broth with varying pH levels (1–10), which was adjusted with acetic acid (99%) and 5 N NaOH. Subsequently, the inoculated broths were incubated at 37°C for 24 h, and growth was monitored with a spectrophotometer (Evolution™ 220 UV-Visible Spectrophotometer, Thermo Scientific™, USA) at 600 nm wavelength against uninoculated broth.

### Biofilm Formation Detection Using the Congo Red Agar Method

The production of biofilm was analyzed according to previously described methods (33). In brief, the isolated probiotic strains were streaked on Mueller–Hinton agar supplemented with 0.8 g/L of Congo red dye (Shanghai Macklin Biochemical Co., Ltd.) and incubated later on at 37°C for 48 h. Black colony presence with dry crystalline consistency showed biofilm production, whereas those with red colonies showed non-biofilm-producing strains.

### Statistical Analysis

All the experiments were conducted in triplicate, and the results obtained were subjected to a one-way analysis of variance (ANOVA) using the Statistical Package for Social Sciences (SPSS) software for Windows (IBM SPSS v20.0, Inc., 2010, Chicago, USA). Differences between means were tested by Tukey's honestly significant difference (HSD) test. A difference was considered to be statistically significant (*P* < 0.05), and the results are presented as mean ± standard error (SE).

## Results

### Identification of Probiotic *Bacillus* Strains

**Figure 1:**
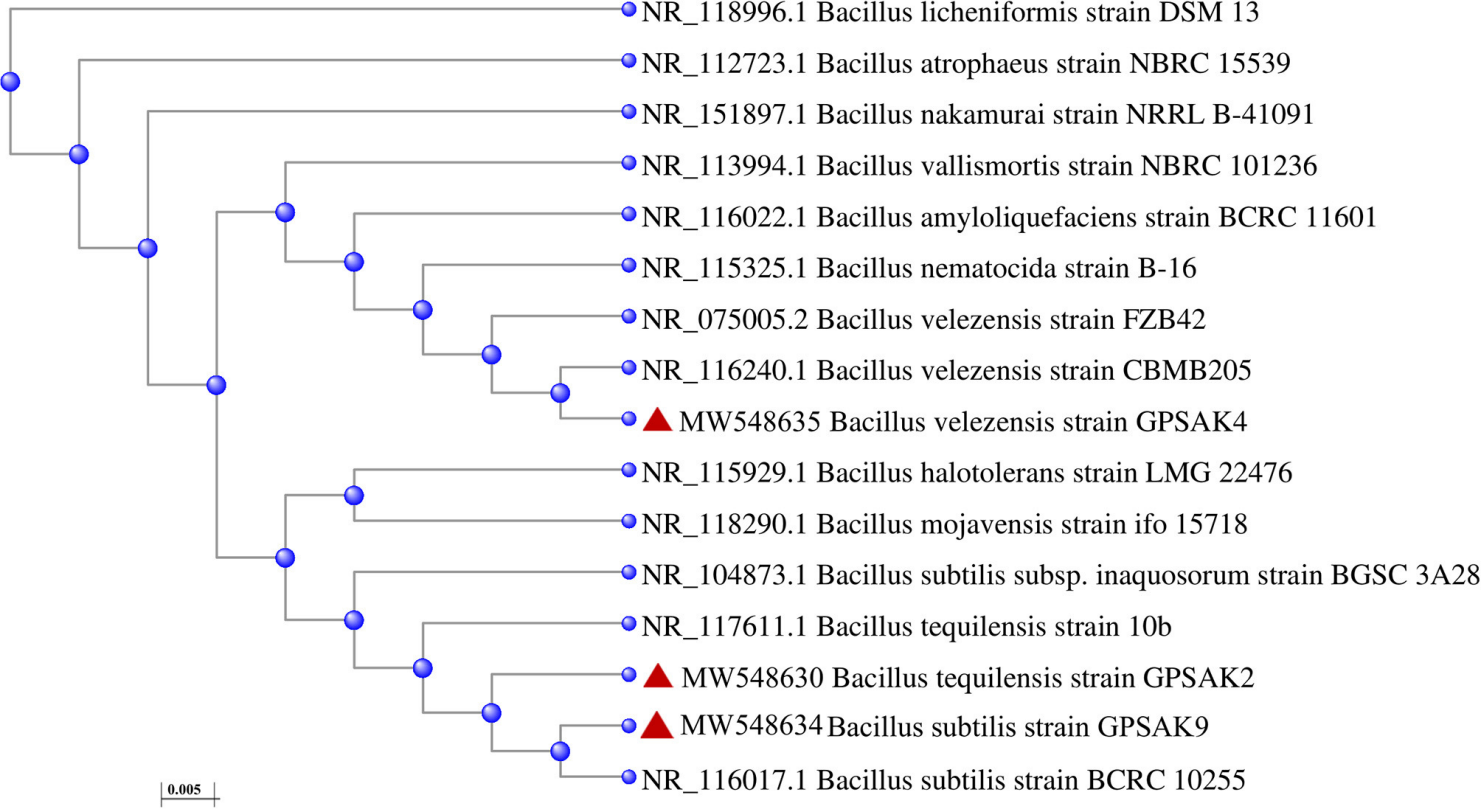
The phylogenetic tree generated by the neighbor-joining method using the NCBI distance tree based on their 16S rRNA sequences. The relationships between three isolated strains (GPSAK2, GPSAK4, and GPSAK9) and other closely related Bacillus species are shown. Numbers before the bacteria species are the accession number in NCBI GenBank.

The three isolated probiotic strains were designated as GPSAK2, GPSAK9, and GPSAK4. These probiotic strains were selected based on their morphological and biochemical characterization as described (see section Morphological and Biochemical Characterization of Isolates). The three isolated strains, GPSAK2, GPSAK4, and GPSAK9, showed close sequence homology (98–99%) to *Bacillus tequilensis, Bacillus velezensis*, and *Bacillus subtilis*, respectively. The phylogenetic tree generated by the NJ method using NCBI Distance tree online tool, as illustrated in Figure 1, established that the isolates GPSAK2, GPSAK4, and GPSAK9 were closest to *B. tequilensis, B*. *velezensis*, and *B*. *subtilis*, respectively. The nucleotide sequences obtained for these three strains have been deposited in the NCBI GenBank database under accession numbers MW548630 (*B. tequilensis* GPSAK2), MW548635 (*B. velezensis* GPSAK4), and MW548634 (*B. subtilis* GPSAK9).

### Morphological and Biochemical Characterization of Isolates

**Figure 2:**
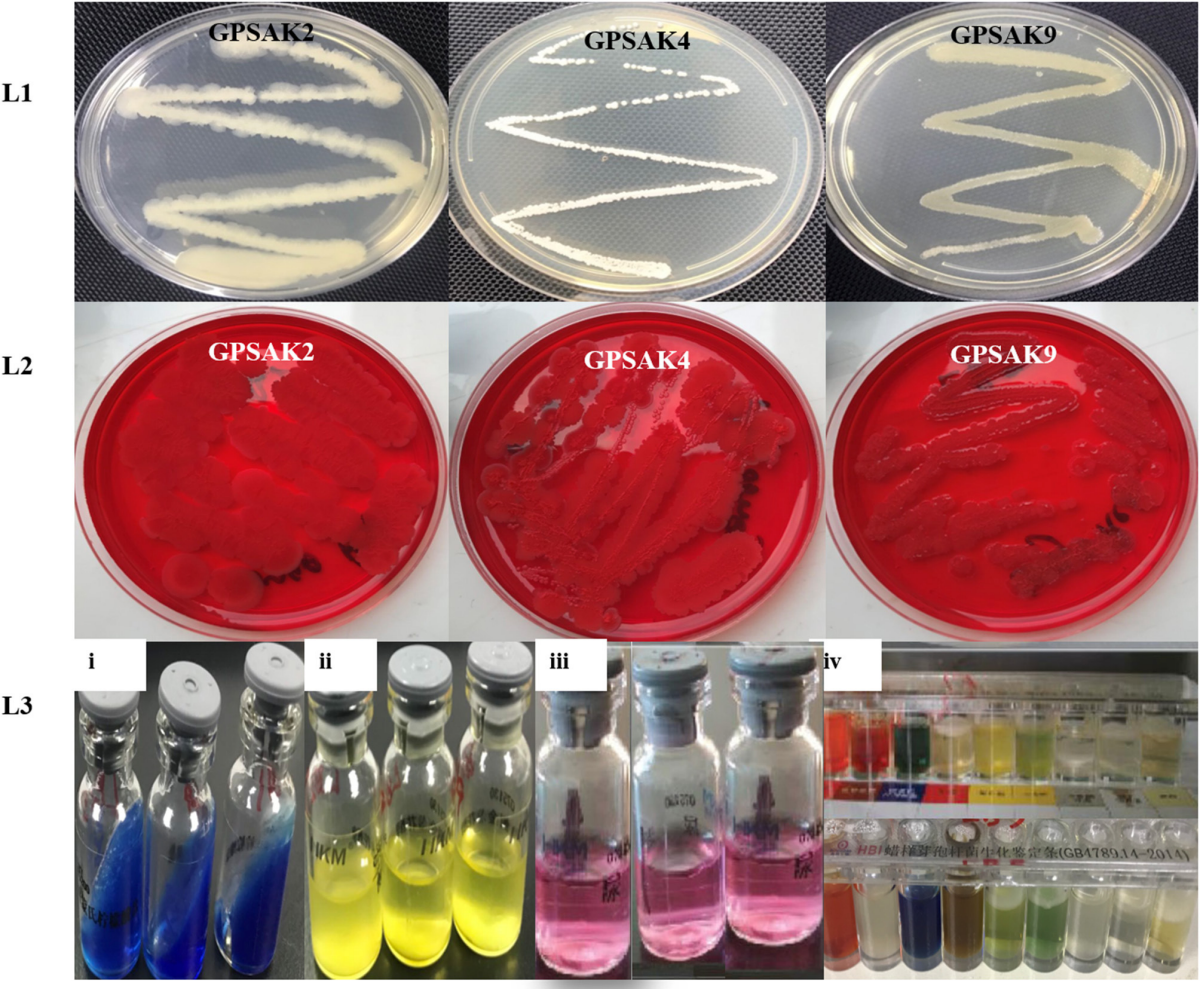
A pictorial overview of the morphological and biochemical characteristics of the three isolated Bacillus strains. Lane 1 (L1): morphology of GPSAK2, GPSAK4, and GPSAK9 strains; Lane 2 (L2): biofilm formation detection of isolates (GPSAK2, GPSAK4, and GPSAK9) using congo red agar method; Lane 3 (L3): results of the biochemical test of isolates (3i: citrated reduction test, 3ii: adonitol test, 3iii: arginine dihydrolase test, 3iv: a confirmatory test using *Bacillus cereus* identification bar).

The morphological and biochemical identification results are summarized in Tables 1, 2, respectively. It showed that all the isolates had an almost similar biochemical characteristics and thus were positive for Gram staining, rhamnose, inositol, sorbitol, adonitol, Simon's citrate, Vorges Proskauer (VP), arginine dihydrolase, spore formation, gelatin liquefaction; capable of producing catalase; aiding in the reduction of nitrate; able to metabolize lactose, starch, and glucose; and able to grow in lysozyme broth. All isolates were noted to reveal negative results for methyl red, urease, hippuric acid, and biofilm production test. GPSAK2 and GPSAK9 showed negative results for mannitol in contrast to the results observed for the GPSAK4 strain. GPSAK4 strain was thereby noted to be halophilic, since it could grow in lysozyme broth and was mannitol positive (Table 2). Figure 2 L1 shows the different morphological characteristics of the isolated strains.

**Table 1:** The morphological characterization of the three probiotic strains isolated from the gut of the hybrid grouper.

**Isolate**	**Form**	**Texture**	**Surface**	**Color**	**Elevation**	**Size**	**Margin**
GPSAK2	Irregular	Rough	Dry	Creamy White	Umbonate	Medium	Undulate
GPSAK4	Circular	Rough	Mucoid	White	Raised	Medium	Entire
GPSAK9	Irregular	Rough	Moist	Creamy Yellow	Crateriform	Medium	Entire

**Table 2:** The biochemical characterization of three probiotic strains isolated from the gut of the hybrid grouper.

**Tests conducted**	**Isolates**			**Tests conducted**	**Isolates**		
	**GPSAK2**	**GPSAK4**	**GPSAK9**		**GPSAK2**	**GPSAK4**	**GPSAK9**
Rhamnose	+	+	+	Hemolysis	γ	α	γ
Sorbitol	+	+	+	Catalase	+	+	+
Inositol	+	+	+	VP	+	+	+
Adonitol	+	+	+	Methyl Red	-	-	-
Simon's citrate	+	+	+	Urease	-	-	-
Lactose fermentation	+	+	+	Gelatin liquefaction	+	+	+
Starch hydrolysis	+	+	+	Hippuric acid	-	-	-
Glucose	+	+	+	Mannitol	-	+	-
Arginine dihydrolase	+	+	+	Gram staining	+	+	+
Nitrate reduction	+	+	+	Biofilm production	-	-	-
Lysozyme broth	+	+	+	Spore formation	+	+	+

*+, positive; –, negative; VP, vorges proskauer*.

### The Growth Pattern of the Three Isolated Strains

**Figure 3:**
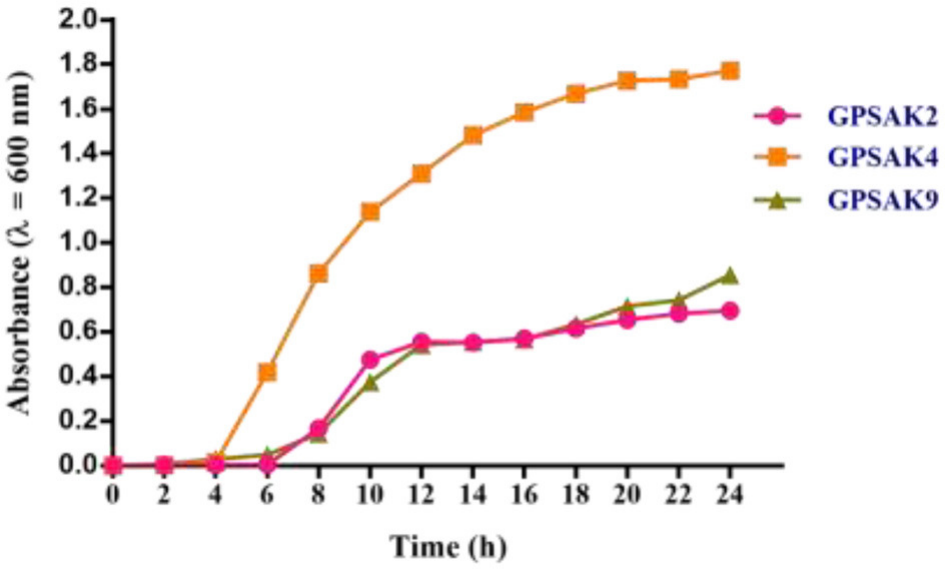
The growth pattern of the three isolated strains measured at an absorbance wavelength of 600 nm.

The bacterial growth pattern of the three isolated strains (GPSAK2, GPSAK4, and GPSAK9) is presented in Figure 3. Although the log phase of strains GPSAK4 and GPSAK9 started approximately at 2 h, that of the GPSAK2 strain started at ~ 6 h after incubation (37°C) with continuous shaking (150 rpm) (Figure 3). In the culture process, the count of the vegetative cells for the GPSAK4 strain was higher within the 24-h culture period than the GPSAK2 and GPSAK9 strains. GPSAK2 strain attained its stationary phase earlier than the others.

### Biosafety Test

Regarding the *in vivo* biosafety test, no pathological symptoms (i.e., gross examination of hemorrhage, edema, lethargic, lesions, and detachment of scales) were observed in the control and experimental fish. Furthermore, there were no recordings of mortalities confirming the non-pathogenic property of the three isolates.

### Antibiotic Susceptibility Test

Table 3 illustrates the results of the antibiotic susceptibility of the selected isolates. Twenty-four (24) antibiotics were tested in this current study. It was observed that all the isolates were highly susceptible (S) to most (19) of the antibiotics. GPSAK9 strain showed resistance (R) to clindamycin, furazolidone, vancomycin, and ampicillin and again showed moderate susceptibility (MS) to sulfamethoxazole and medecamycin antibiotics, whereas that of GPSAK2 showed MS to only furazolidone.

**Table 3:** Susceptibility of isolates (mm) to antibiotics.

**Antibiotics**	**μg/disc**	**Isolates**			**Antibiotics**	**μg/disc**	**Isolates**		
		**GPSAK2**	**GPSAK4**	**GPSAK9**			**GPSAK2**	**GPSAK4**	**GPSAK9**
Ampicillin	10	S	S	R	Minocycline	30	S	S	S
Penicillin	10	S	S	S	Ofloxacin	5	S	S	S
Kanamycin	30	S	S	S	Norfloxacin	10	S	S	S
Ceftriaxone	30	S	S	S	Doxycycline	30	S	S	S
Chloramphenicol	30	S	S	S	Neomycin	30	S	S	S
Erythromycin	15	S	S	S	Gentamicin	10	S	S	S
Clindamycin	2	S	S	R	Tetracycline	30	S	S	S
Furazolidone	300	MS	S	R	Carbenicillin	100	S	S	S
Polymyxin B	300	S	S	S	Midecamycin	30	S	S	MS
Vancomycin	30	S	S	R	Ciprofloxacin	5	S	S	S
Sulfamethoxazole	27.5	S	S	MS	Piperacillin	100	S	S	S
Amikacin	30	S	S	S	Cefoperazone	75	S	S	S

*S, susceptible; R, resistant; MS, moderately susceptible*.

### Bile Salt Tolerance

**Figure 4:**
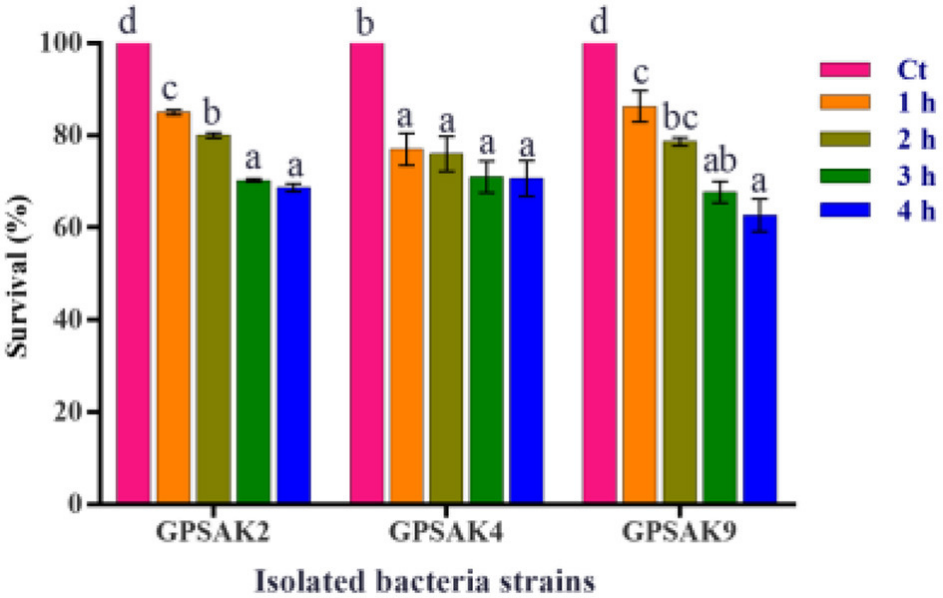
Bile tolerance of the three *Bacillus* strains isolated from the gut of hybrid grouper. Values are presented as mean ± SE. Significant differences are indicated by different letters (*P* < 0.05). Ct represents the control or time at 0 h.

The three isolated strains' tolerance and survivability to 0.5% bile salt were monitored by counting the number of CFUs after 1, 2, 3, and 4 h of exposure, which was then expressed in percentage. The results demonstrated that more than 50% of the isolates survived after 4 h of exposure (Figure 4). It was observed that increasing the culture hours during bile salt exposure revealed a decreasing trend concerning the survival percentages of the isolated strains' CFU count. There were significant reductions (*P* < 0.05) in percentage survival of all isolates after 1 h bile salt exposure. While no significant differences (*P* > 0.05) were observed between the hours (1, 2, 3, and 4 h) exposed to bile salt in the GPSAK4 strain, that of the GPSAK2 and GPSAK9 strains revealed significant reductions (*P* < 0.05) in the percentage survival of the CFU counts.

### High-Temperature Resistance Test

**Figure 5:**
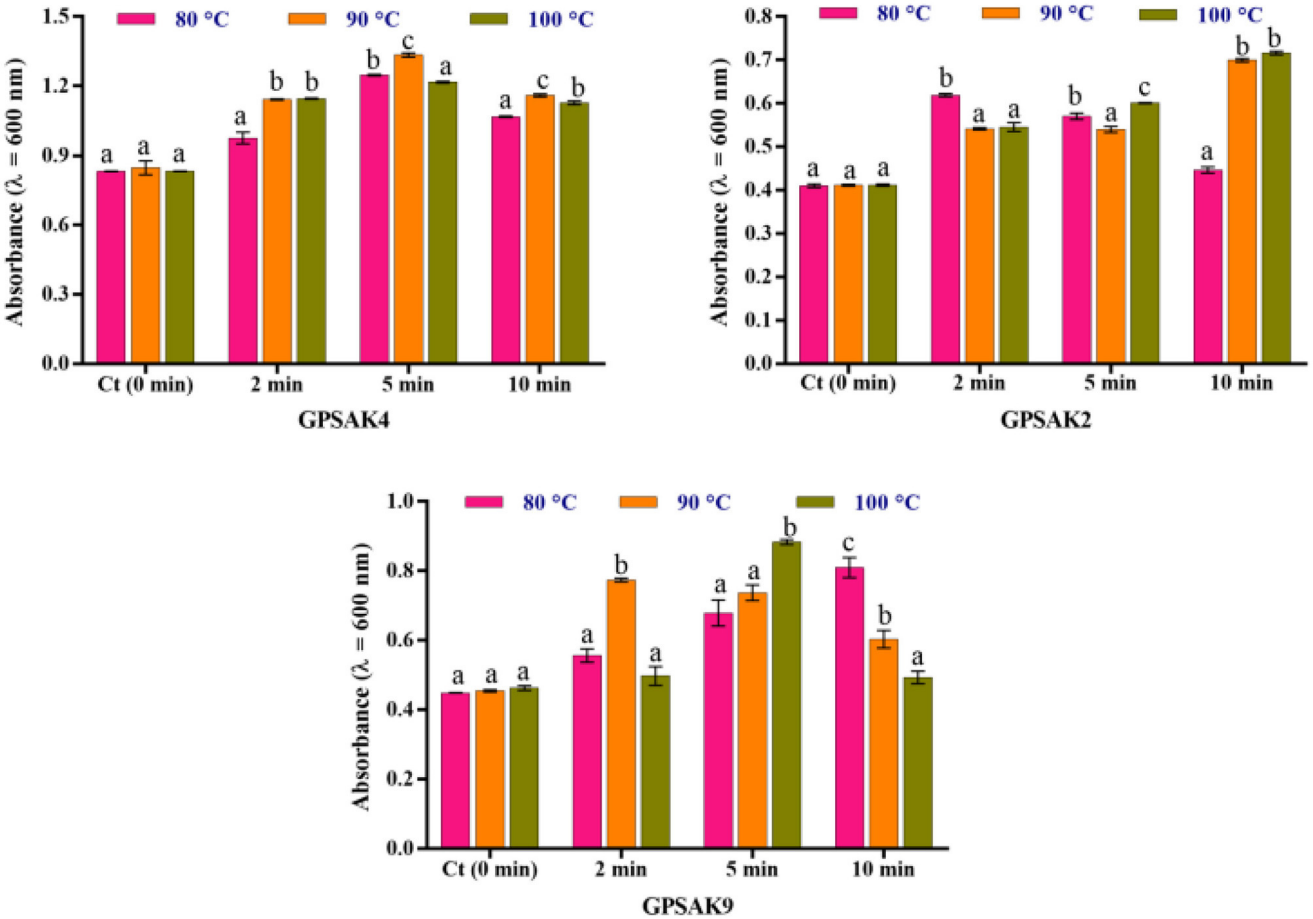
The resistance of the three strains isolated from the gut of the hybrid grouper to high temperature. Values are presented as mean ± SE. Significant differences are indicated by different letters (*P* < 0.05).

Figure 5 illustrates the results obtained after isolates were exposed to different temperature conditions (80, 90, and 100°C) at different time points (2, 5, and 10 min) of which the three isolates gave promising results. After the trial, higher OD growth signifying higher growth of vegetative cell counts was observed in all the isolates exposed to the varying temperatures compared to the control (isolates without exposure to the higher temperatures). There were significant differences (*P* < 0.05) at the different times of exposure among the different temperatures (Figure 5). In the GPSAK2 strain, there were significantly high (*P* < 0.05) OD values witnessed when heated at (i) 80°C for 2 min compared to when heated at 90 and 100°C; (ii) 100°C for 5 min compared to when heated at 80 and 90°C; and (iii) both 90 and 100°C for 10 min compared to when heated at 80°C. For the GPSAK4 strain, a significant increase (*P* < 0.05) in the OD values was observed when heated at (i) both 90 and 100°C for 2 min compared to when heated at 80°C; (ii) 90°C for 5 min compared to when heated at 80 and 100°C; and (iii) 90°C for 10 min compared to when heated at 80 and 100°C. The GPSAK9 strain, on the other hand, showed significantly high (*P* < 0.05) OD values when heated at i) 90°C for 2 min compared to when heated at 80 and 100°C; (ii) 100°C for 5 min compared to when heated at 80 and 90°C; and (iii) 80°C for 10 min compared to when heated at 90 and 100°C.

### Compatibility of the Three Isolates

In the current study, when the isolated strains were characterized for their compatibility, no definite sign of suppression of the three bacterial isolates was observed on each other, suggesting that they were compatible.

### Antimicrobial Properties

**Figure 6:**
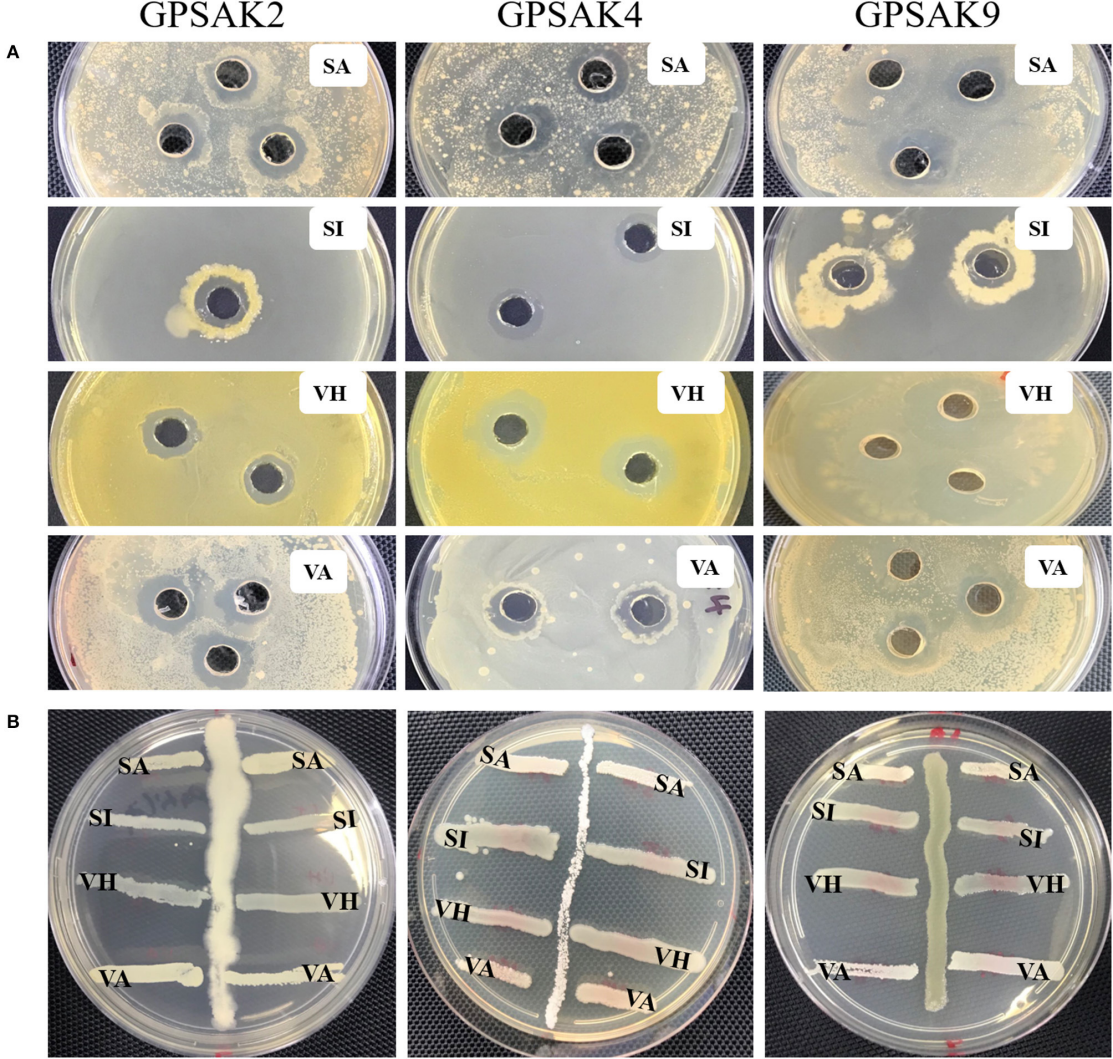
Pictorial view showing the growth inhibition zones of the isolated strains GPSAK2, GPSAK4, and GPSAK9 against the four pathogens (SA, *Streptococcus agalactiae*, SI, *Streptococcus iniae*, VH, *Vibrio harveyi*, and VA, *Vibrio alginolyticus*) using the **(A)** agar well-diffusion method and **(B)** cross streak method.

In the current study, the three isolated bacteria were assessed for their antimicrobial properties against the four fish pathogens, *viz. S. agalactiae, S. iniae, V. harveyi*, and *V. alginolyticus*. There were promising results for the isolates in the cross streaking and agar well-diffusion method (Figure 6). It was revealed at the end of the trial that the three isolates showed inhibition against the four pathogenic strains (Table 4). The GPSAK9 strain exhibited higher antimicrobial activity compared with the GPSAK2 and GPSAK4 strains.

**Table 4:** Antimicrobial activity of the three isolates against the selected fish pathogens.

**Name of fish pathogen**	**Isolates**		
	**GPSAK2**	**GPSAK4**	**GPSAK9**
*Streptococcus agalactiae*	++	+++	++++
*Streptococcus iniae*	+	++	+
*Vibrio harveyi*	++	+++	++++
*Vibrio alginolyticus*	+++	+	+++

*Values were calculated as inhibition zone diameter minus well diameter (mm). Where “+” indicates zone of inhibition between 1 and 2 mm, “++” indicates zone of inhibition between 3 and 4 mm, “+++” indicates zone of inhibition between 5 and 6 mm, and “++++” indicates zone of inhibition between 7 and 8 mm*.

### Screening of *Bacillus* Strains for Auto-Aggregation and Cell Surface Hydrophobicity Properties

**Figure 7:**
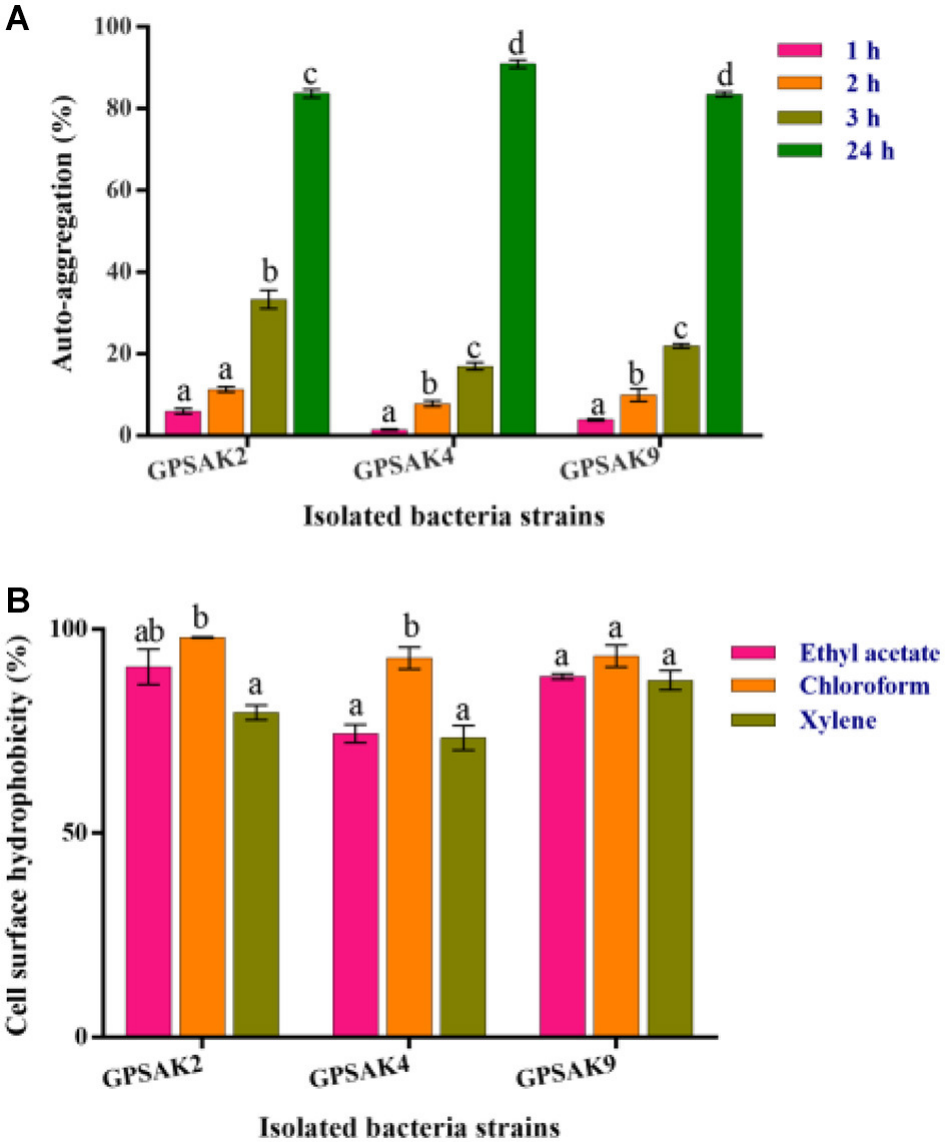
**(A)** Auto-aggregation ability of the isolates after 24 h, and **(B)** Cell surface hydrophobicity (%) of the isolated *Bacillus* strains with ethyl acetate, chloroform, and xylene solvents.

Figure 7A shows the auto-aggregation ability of the three isolates. Auto-aggregation ability assays strongly correlate with cell adhesions to the digestive tract. The results revealed that all the isolates (GPSAK2, GPSAK4, and GPSAK9) witnessed low cell adhesion ability (< 40%) at the first 3 h. Nonetheless, after 24 h, the cell adhesion of the GPSAK2, GPSAK4, and GPSAK9 strains increased significantly (*P* < 0.05) to 83.7, 90.8, and 83.5%, respectively.

The adhesion of the isolated strains (GPSAK2, GPSAK4, and GPSAK9) to ethyl acetate, chloroform, and xylene solvents was again tested to determine adhesion capabilities of bacteria to cell surfaces of which the results are illustrated in Figure 7B. It was observed that the cell surface hydrophobicity of the GPSAK2 and GPSAK4 isolates to ethyl acetate and xylene was significantly lower (*P* < 0.05) than that of chloroform. The GPSAK2 strain showed much higher hydrophobicity (90.8 and 98%) with ethyl acetate and chloroform, illustrating that its bacterial adhesion to hydrocarbon compound is better than that of GPSAK4 (77.4 and 92.9%) and GPSAK9 (88.4 and 93.4%). However, concerning the results of xylene, the GPSAK9 strain revealed the highest hydrophobicity percentage (87.5%) compared to the GPSAK2 (79.6%) and the GPSAK4 (73.4%) strains. No significant difference (*P* > 0.05) was witnessed in the solvents of the GPSAK9 strains.

### Hemolytic Activity

Concerning the hemolytic activities, GPSAK2 and GPSAK9 exhibited γ-hemolysis, whereas that of the GPSAK4 strain exhibited α-hemolysis (see Table 2 in section Morphological and Biochemical Characterization of Isolates).

### Determination of Optimal Growth and pH of the Three Isolated Probiotics

**Figure 8:**
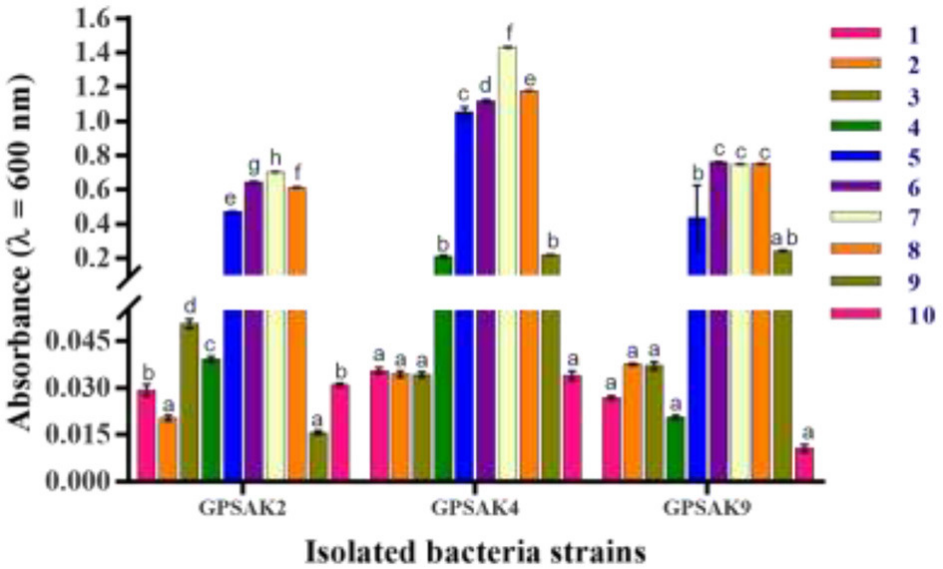
Bacterial growth at different pH (1.0–10.0) levels measured at an absorbance wavelength of 600 nm. Values are presented as mean ± SE. Significant differences are indicated by different letters (*P* < 0.05).

All isolates, after different pH exposures, gave promising tolerance results. Although there was an irregular growth pattern, there was a gradual increase in vegetative cell growth within the pH range of 1.0–7.0 (GPSAK2 and GPSAK4 strains, optimum growth found to be at 7.0 pH) and 1.0–8.0 (GPSAK9 strain, optimum growth found between 6.0 and 8.0 pH). No significant difference (*P* > 0.05) in vegetative cell growth of strain GPSAK9 was observed between pH 6.0 and 8.0. Decreased growth was observed as pH increased from 7.0 to 10.0 (for both GPSAK2 and GPSAK4 strains) and 8.0 to 10.0 (for GPSAK9 strain), implying that the isolates could survive in extreme alkaline and acidic conditions. There were significant differences (*P* < 0.05) displayed in the isolates at different pH levels (Figure 8).

### Biofilm Formation Detection Using the Congo Red Agar Method

Biofilm formation detection was conducted using the Congo red agar method. All the isolates indicated negative biofilm production at the end of the test, as none of them formed black colonies [Table 2 and Figure 2 (L2)].

## Discussion

The management of diseases efficiently in aquaculture is crucial for the fruitful production of aquatic animals and the aquaculture industry's sustainability (31, 54). The wide and inappropriate usage of antibiotics has resulted in severe biological and ecological concerns, especially resulting in the emergence of antibiotic-resistant bacteria (13, 14). Probiotics, known as beneficial microbes, are proposed as an effective and eco-friendly alternative to antibiotics due to their enormous health benefits to host organisms and their resistive abilities against pathogens (55). *Bacillus* spp. have been reported as one of the most outstanding probiotics due to the very encouraging properties they have compared to others (20, 31–35). Correspondingly, several reports have highlighted the beneficial effects of *Bacillus* spp. in grouper aquaculture production (56–59). Ramesh et al. (40), in their work, asserted that *Bacillus* spp. isolated from the intestine of healthy fish are regarded as the best source for bacterial isolation to help control fish diseases consistent with our source of isolated strains.

In the current study, three *Bacillus* species *viz. B*. *tequilensis* GPSAK2 (MW548630), *B*. *velezensis* GPSAK4 (MW548635), and *B*. *subtilis* GPSAK9 (MW548634) were isolated from the intestine of hybrid grouper and their potential probiotic abilities assessed using *in vitro* methods. The identification of the isolated strains (GPSAK2, GPSAK4, and GPSAK9) was achieved using morphological characteristics and biochemical tests and further confirmation tests done by 16S rRNA gene sequencing. It was found that all isolates showed properties of utilizing a wide range of carbon sources such as rhamnose, inositol, sorbitol, adonitol, citrate, gelatin, lactose, starch, and glucose, in addition to amino acid arginine. Having these properties suggests that the isolates could be helpful in the digestion and hydrolysis of carbohydrates and amino acid, respectively, thus could be used as probiotics and for the production of value-added products in the food industries as previously reported in other researches (33, 40, 52, 60).

For a bacterium to be regarded as having probiotic properties, it must not cause any adverse effects, which can lead to disease infections; thus, it must be safe to the host organism. Again, it must have the ability of not harboring acquired and transferable antibiotic resistance genes (13, 61). In this study, the biosafety test conducted revealed that all the isolates were safe for hybrid grouper. Also, the antibiotic susceptibility test illustrated that out of the 24 antibiotics tested; all the isolates were highly susceptible to 19 of them, namely, penicillin, kanamycin, ceftriaxone, chloramphenicol, erythromycin, clindamycin, polymyxin B, amikacin, minocycline, ofloxacin, norfloxacin, doxycycline, neomycin, gentamicin, tetracycline, carbenicillin, ciprofloxacin, piperacillin, and cefoperazone, similar to previously reported works (29, 60). GPSAK9 strain showed resistance to clindamycin, furazolidone, vancomycin, and ampicillin and again showed moderate susceptibility to sulfamethoxazole and medecamycin antibiotics, whereas that of GPSAK2 showed moderate susceptibility to only furazolidone. The resistance of *B*. *subtilis* strains to clindamycin, vancomycin, and ampicillin antibiotics reported previously was attributed to the presence of a naturally resistant gene of the isolated strains (62). Thus, although we did not evaluate such naturally occurring resistant genes, we believe that the presence of such genes might have caused the discrepancies of antibiotic resistance tests among the isolated *Bacillus* strains in the current study.

*Bacillus* spp. unlike other probiotics are spore formers, making them more tolerant to extreme heat (29, 63) and tolerant to low pH and a high percentage of bile concentration (64), making them survive and grow in the fish gut (65). To survive and colonize the host organism's gut to release beneficial effects, a probiotic bacterium must possess the ability to tolerate low pH (gastric) and a high percentage of bile concentration (66, 67). According to Garcia-Ruiz et al. (68), as the bile concentration ranges from 0.3 to 0.5% in the intestine of humans, it will be very beneficial to have isolated species survive in such percentage range of bile. As a good biotechnological attribute, the probiotic must also have the ability to withstand extreme heat conditions to stay viable, such as after feed making, since animal feed mostly depends on heat to attain high palatability and kill pathogenic cells (29). It is worth mentioning that all strains (GPSAK2, GPSAK4, and GPSAK9) isolated in the current study displayed good sporulation efficiency, which metamorphosed into their capacity to withstand 0.5% bile concentration, tolerate pH as low as 1, and survive highly after heat treatment in comparison to the control. It could consequently be presumed that the higher temperatures (80, 90, and 100°C) activated the bacterial strains (69), hence observing the increase in growth after heat treatment. Correspondingly, other studies support the sporulation efficiency of *B*. *tequilensis* (70, 71), *B*. *velezensis* (60, 72, 73), and *B*. *subtilis* (39, 41, 60, 63). The higher viability efficiency exhibited by the isolated strains in the present study depicts the good potentials they have when it comes to being used as probiotics in the feed.

The most substantial evidence in favoring an isolated bacterium for it to be chosen as a probiotic hinges on its antagonistic properties against pathogens. Earlier reports have demonstrated the antimicrobial properties of *Bacillus* species against pathogenic bacteria. In this study, all the isolated strains showed great antagonistic effects against the four selected pathogenic bacteria, *viz. S. agalactiae, S. iniae, V. harveyi*, and *V. alginolyticus*. There were clear zones of inhibition appearing in the pathogenic culture broth, which meant that the isolated bacterial strains' secretion could constrain potential pathogens' growth. Several fish diseases reported to be hampering fish production are noted to be caused by *Streptococcus* spp. (74) and *Vibrio* spp. (75, 76). For example, in 2012, there was severe *S*. *agalactiae* infection in giant grouper (*E*. *lanceolatus*) and other wild fish in Australia (77). Hence, the results obtained suggest that the three isolated *Bacillus* strains GPSAK2, GPSAK4, and GPSAK9 could help fight against such fish diseases and in turn aid in the sustenance of the aquaculture industry.

Another useful probiotic attribute commonly included as an *in vitro* test is colonizing the intestinal mucosa. That is, the purported potential bacteria must have the ability to adhere to the mucosal surface and epithelial cells (78) and also block the adhesion of the pathogenic bacteria, so that the work of balancing the intestinal microbial composition (79) and the enhancement of the host's immune system can be achieved (80). The use of auto-aggregation and hydrophobicity are indirect methods for testing probiotics' cell adhesion ability (81). It was revealed that the GPSAK2 strain showed much higher percentage hydrophobicity (90.8 and 98%) with ethyl acetate and chloroform, illustrating high bacterial adhesion to hydrocarbon compound than GPSAK4 (77.4 and 92.9%) and GPSAK9 (88.4 and 93.4%). However, concerning the results of xylene, the GPSAK9 strain revealed the highest hydrophobicity percentage (87.5%) compared to the GPSAK2 (79.6%) and the GPSAK4 (73.4%). No significant difference (*P* > 0.05) was witnessed in the solvents of the GPSAK9 strains. The observations of the percentage hydrophobicity were comparatively higher than the results obtained by Patel et al. (47) and Lee et al. (52) but was quite similar to previous work (60), which illustrates higher electron acceptance (ethyl acetate) and donation (chloroform) (82). Moreover, a strong correlation exists between auto-aggregation and the adhesion of cells to the digestive tract, which is an indispensable characteristic for a good bacterium (83). Our study demonstrated that the isolated strains GPSAK2, GPSAK4, and GPSAK9 witnessed high auto-aggregation of 83.7%, 90.8%, and 83.5%, respectively, corroborating with previous findings (60, 84).

Hemolytic, compatibility, and biofilm formation tests are also considered very important in identifying a potential probiotic bacterium *in vitro*. Hemolysin produced by a pathogen is noted to lyse host cells to release iron-containing compounds such as hemoglobin, which is beneficial for the growth of bacteria in the host organism (48, 85). While the test results of β-hemolysis are considered harmful, no- or γ-hemolysis and α-hemolysis are regarded safe (51). In the current study, γ-hemolysis was exhibited by the GPSAK2 and GPSAK9 strains, whereas α-hemolysis was the result obtained for the GPSAK4 strain. The findings corroborated with those of Kavitha et al. (33) and Lee et al. (52). Compatibility tests are mostly conducted to ascertain whether the isolated strains can be used as multispecies probiotics or not. Saarela et al. (86) in their findings demonstrated that food produced from mono-species probiotics had an acidic and sour taste. The present study illustrated that all three isolates were compatible with each other to be used as multispecies, agreeing with the work of Rajyalakshmi et al. (49). Regardless of the benefits linked with biofilm formation (87, 88), biofilm tests for bacteria are vital to public health, since biofilm-forming bacteria are noted to display reduced susceptibility to antimicrobial agents (89). The current study showed that all isolates (GPSAK2, GPSAK4, and GPSAK9) were negative for forming biofilm tests, similar to the findings of Kuebutornye et al. (60). The experiment conducted by Kavitha et al. (33) sought to suggest that only one was positive for biofilm out of the three isolates obtained.

## Conclusion

The study demonstrated that all the three strains, GPSAK2, GPSAK4, and GPSAK9, isolated from the intestine of hybrid grouper (*E*. *fuscoguttatus*♀ × *E*. *lanceolatus*♂), possess desirable potential probiotic characteristics based on their high survivability after treatment with heat, broad antimicrobial activity, non-hemolytic nature, as well as their safety confidence such as their antibiotic susceptibility. Taking all of the results into account, these bacterial strains demonstrate that they could serve as great probiotic potential for use in aquaculture. Nevertheless, additional *in vitro* or *in vivo* experiments should be performed to ascertain whether they can be approved for application in the aquaculture environment, especially in grouper culture.

## Data Availability Statement

The datasets presented in this study can be found in online repositories. The names of the repository/repositories and accession number(s) can be found at: https://www.ncbi.nlm.nih.gov/genbank/, (MW548630), https://www.ncbi.nlm.nih.gov/genbank/, (MW548635), https://www.ncbi.nlm.nih.gov/genbank/, (MW548634).

## Ethics Statement

The animal protocol used in the present study was approved by the ethics review board of Guangdong Ocean University, and all procedures were performed in accordance with the standards of the National Institutes of Health Guidelines for the Care and Use of Laboratory Animals and relevant Chinese policies.

## Author Contributions

KA and X-hD designed the study. KA conducted the study, analyzed the data, and wrote the manuscript. B-pT participated in the interpretation of results. SZ, FKAK, S-yC, Q-hY, and H-yL purchased the reagent supplies. KA, H-tZ, and Y-zY submitted sequences and revised the manuscript. All authors have read and approved the final version of the manuscript.

## Conflict of Interest

The authors declare that the research was conducted in the absence of any commercial or financial relationships that could be construed as a potential conflict of interest.
